# The use of proton pump inhibitors decreases the risk of diabetes mellitus in patients with upper gastrointestinal disease: A population-based retrospective cohort study

**DOI:** 10.1097/MD.0000000000004195

**Published:** 2016-07-18

**Authors:** Hsiu-Chen Lin, Yu-Ting Hsiao, Hsiu-Li Lin, Yow-Shieng Uang, Hui-Wen Cheng, Ying Wang, Li-Hsuan Wang

**Affiliations:** a Department of Pediatrics, School of Medicine, College of Medicine, Taipei Medical University, Taipei; b Department of Laboratory Medicine, Taipei Medical University Hospital, Taipei; c School of Public Health, College of Public Health and Nutrition, Taipei Medical University, Taipei; d School of Pharmacy, College of Pharmacy, Taipei Medical University, Taipei; e Graduate Institute of Biomedical Informatics, College of Medical Science and Technology, Taipei Medical University, Taipei; f Department of Neurology, Cathy General Hospital, Sijhih Branch, New Taipei City; g Department of Pharmacy, Taipei Medical University Hospital, Taipei, Taiwan.

**Keywords:** cohort study, defined daily dose, diabetes mellitus, proton pump inhibitors, risk

## Abstract

**Objectives:**

The aim of this study was to investigate the effects of proton pump inhibitors (PPIs) on the risk of diabetes mellitus (DM) among patients with upper gastrointestinal disease (UGID).

**Methods:**

This was a retrospective cohort study with a follow-up period of 5 years. We identified 388,098 patients who were diagnosed with UGID between 2000 and 2006 from the Longitudinal Health Insurance Database of the Taiwan National Health Insurance program. We used Cox proportional hazard ratio (HR) to compare the risk of DM between UGID patients received PPIs and those did not receive PPIs. HRs were adjusted for possible confounders, including age, sex, hypertension, gout and/or hyperuricemia, coronary artery disease, stroke, pancreatitis, hyperlipidemia, obesity, H2-blocker use, and clozapine or olanzapine use. The dose-related effects of PPIs on the risk of DM were evaluated according to the defined daily dose (DDD).

**Results:**

The adjusted HR was 0.80 (95% CI, 0.73–0.88) for the study group (UGID patients with PPIs) compared with comparison group I (UGID patients without PPIs). Among patients who used PPIs, those older than 60 years of age had a lower risk of DM (HR, 0.73; 95% CI, 0.63–0.83) than those younger than 40 years. Additionally, the effect of PPIs was significantly dose-dependent (*P* for trend <0.001). Patients with UGID who received >540 DDDs of PPIs exhibited the greatest reduction in the risk of DM.

**Conclusions:**

Our results demonstrated a decreased risk of DM in UGID patients who used PPIs; the risk appeared to be significantly dose-dependent.

## 1 Introduction

Diabetes mellitus (DM) is a worldwide epidemic and the number of people with DM has more than doubled globally in the past 3 decades.^[1]^ Type 2 DM (T2DM) is caused by peripheral insulin resistance and is usually characterized by β-cell hyperplasia and hyperinsulinemia.^[2]^ Proton pump inhibitors (PPIs) are widely used for the treatment of gastric acid-related diseases such as peptic ulcer disease and gastroesophageal reflux disease.^[3,4]^ PPIs block the last enzyme in the gastric acid secretion system and, consequently, decrease gastric acid secretion and increase the blood concentration of the hormone gastrin.^[5,6]^ Some in vitro studies have demonstrated that gastrin induces β-cell neogenesis from pancreatic exocrine duct cells and increases β-cell mass.^[7,8]^ Several studies have shown that treatment with gastrin can induce the formation of new β-cells under various conditions in animal models.^[9,10]^ Retrospective studies in adults with DM have shown that patients who receive PPIs achieve better glycemic control than patients without receiving PPIs.^[11,12]^ Singh et al^[13]^ designed a randomized, double-blind, placebo-controlled study to evaluate the effect of pantoprazole therapy on glucose-insulin homeostasis in patients with T2DM; the results showed significantly reduced HbA1c levels and increased gastrin levels. Therefore, we hypothesized that PPIs can induce the formation of new β-cells and, consequently, reduce the risk of DM in patients with upper gastrointestinal disease (UGID). Currently, insufficient clinical data exist regarding the effect of PPIs on DM risk, especially among Asian populations, and available studies have not provided a clear analysis of the effect of PPI dose on DM risk reduction. Therefore, we conducted a hypothesis-generating, retrospective study in a Taiwanese population to assess the risk of DM development among UGID patients treated with PPIs.

## 2 Materials and methods

### 2.1 Data sources

This study was a retrospective cohort study. The study samples were retrieved from the Longitudinal Health Insurance Database (LHID), which consisted of 5% samples (about 1,000,000 subjects) of the population included in the Taiwan National Health Insurance (NHI) program. The NHI program was an insurance system and covered for more than 99% of the national population in Taiwan and provided for research purposes. No significant differences in the distribution of age and gender were found between the patients in the sample group and the original population. The LHID contained all medical claimed data for approximately 1,000,000 subjects from 2000 to 2011. It included diagnosis codes, drug prescriptions, hospital visits, including detailed clinical and demographic information of all hospital admissions and ambulatory visits. This study was exempt from full review by the Institutional Review Board of Taipei Medical University because the identification numbers of all of the individuals in the NHRI database were encrypted to protect the privacy of the individuals.

### 2.2 Study sample

For the study cohort, we identified 388,098 patients who were newly diagnosed with UGID (ICD-9-CM codes 530–536, which included diseases of the esophagus, stomach, and duodenum) during an ambulatory care visit between January 1, 2000 and December 31, 2006. Three separate, consecutive diagnoses were required to increase the validity of the diagnosis. For each patient, we assigned the first ambulatory care visit for the treatment of UGID as the index date. We also identified 415,362 patients without UGID (non-UGID patients) who received care between January 1, 2000 and December 31, 2011.

The LHID also provides information on medical orders during ambulatory care visits and hospital admissions. We reviewed this data and determined which subjects had ever filled prescriptions for PPIs during the 5 years after their respective index dates. We classified UGID patients into 2 groups: those who received PPIs (n = 87,679) and those who did not receive PPIs (n = 250,419). In the group of UGID patients who received PPIs, we excluded patients who had been diagnosed with DM and prescribed PPIs before the index date. We also excluded UGID patients who received PPIs after December 31, 2006 and those with fewer than 90 daily doses of PPIs within the first 180 days after the first administration of PPIs.

Finally, we selected 7384 UGID patients who received PPIs as the study group. We also selected 14,768 UGID patients without PPI use as comparison group I. Each patient in the study group was matched to 2 UGID patients without PPI use by age, sex, and index year. Next, we selected 29,536 non-UGID patients as comparison group II. Each patient in comparison group I was matched to 2 non-UGID patients by age, sex, and index year. All of the subjects were followed for 5 years or censored at the date of DM diagnosis.

### 2.3 Dosage of PPI

Complete information about all prescriptions of PPIs was extracted from the NRI prescription database. Data collected included the date of prescription, the daily dose, and the number of days supplied. For the PPIs-treatment group, we calculated the total dosage prescribed during the follow-up period. The defined daily dose (DDD) recommended by the World Health Organization of 20 mg per day was used to quantify omeprazole and rabeprazole usage; 30 mg per day for lansoprazole and esomeprazole usage and 40 mg per day for pantoprazole usage.

### 2.4 Outcome measurement and confounding factors

Each patient was followed for 5 years or until DM was diagnosed, whichever occurred first. The primary outcome was development of DM and the secondary outcome was dose effect of PPIs on the risk of DM. DM was diagnosed according to ICD-9 code 250.0. The measured outcome of DM diagnosis of patients was those with DM diagnosis at least 2 times and with following HbA1C test. We adjusted the risk of DM development for possible confounding factors, including hypertension, gout and/or hyperuricemia, coronary artery disease (CAD), stroke, pancreatitis, hyperlipidemia, obesity, H2-blocker use, and clozapine or olanzapine.

### 2.5 Statistical analysis

Statistical analyses were performed using SAS version 9.1 (SAS System for Windows, Version 8.2, Cary, NC). Student *t* test and Pearson *χ*^2^ test were applied to evaluate differences in sociodemographic characteristics, such as age and sex, and comorbidities among the study cohort and the comparison cohorts. Cox proportional hazard ratios (HRs) were used to estimate HRs and 95% confidence intervals (CIs). Onset time of DM among different DDD groups was evaluated by Student *t* test. The Kaplan–Meier method and log-rank test were used to examine the differences in 5-year DM-occurrence rates between the study and comparison cohorts. All tests were 2-tailed, and *P* values less than 0.05 were considered significant.

## 3 Results

**Figure 1:**
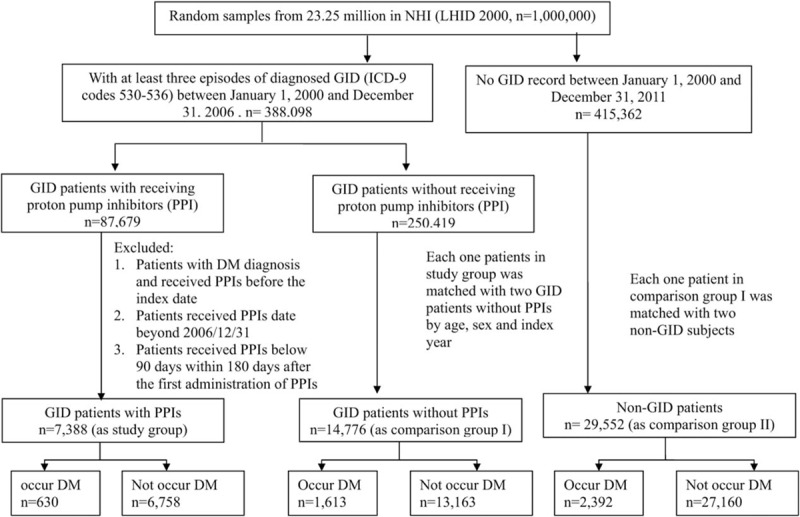
Flow chart of the selection of the study group and 2 matched comparison groups.

Figure 1 illustrates the enrollment of the study group and the 2 comparison groups. All groups were matched for age, sex, and the year of index date.

Table 1 lists the demographic characteristics of the 3 groups. The sex and age distributions were similar among the groups. The mean age of the entire cohort was 55.3 ± 16.96 years and nearly 60% of the subjects were male. The prevalences of comorbid diseases, including hypertension, gout and/or hyperuricemia, CAD, stroke, pancreatitis, and hyperlipidemia, were higher in the study group than in the 2 comparison groups.

**Table 1:**
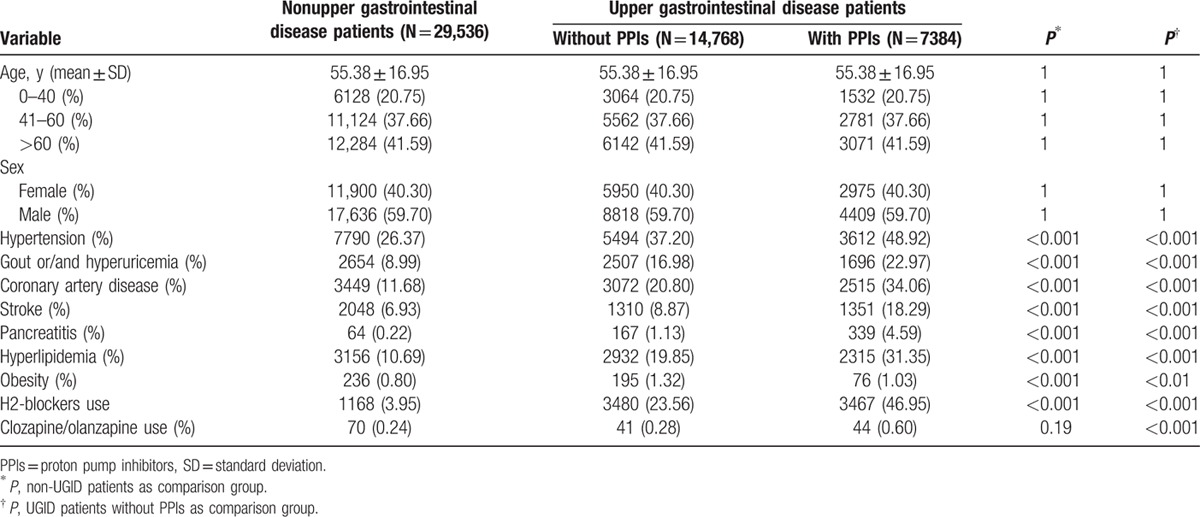
Demographic characteristics of patients with upper gastrointestinal disease (UGID) and without UGID (non-UGID).

We assessed the crude HRs and adjusted HRs for the risk of DM during the 5-year follow-up period between comparison group I and II and between the study group and comparison group I. The adjusted HR for the risk of DM for UGID patients without PPI use (comparison group I) was 1.42 (95% CI, 1.33–1.52) compared with non-UGID patients (comparison group II). The adjusted HR for UGID patients with PPI use (study group) was 0.80 (95% CI, 0.73–0.88) compared with UGID patients without PPI use (comparison group I). Additionally, the adjusted HR for UGID patients with PPI use (study group) was 0.87 (95% CI, 0.78–0.97) compared with non-UGID patients without PPI use (comparison group II).

These results demonstrated that UGID patients had an increased risk of DM after adjustment for various potential confounders. We also observed that UGID patients who received PPIs had a decreased risk of DM (Table 2).

**Table 2:**
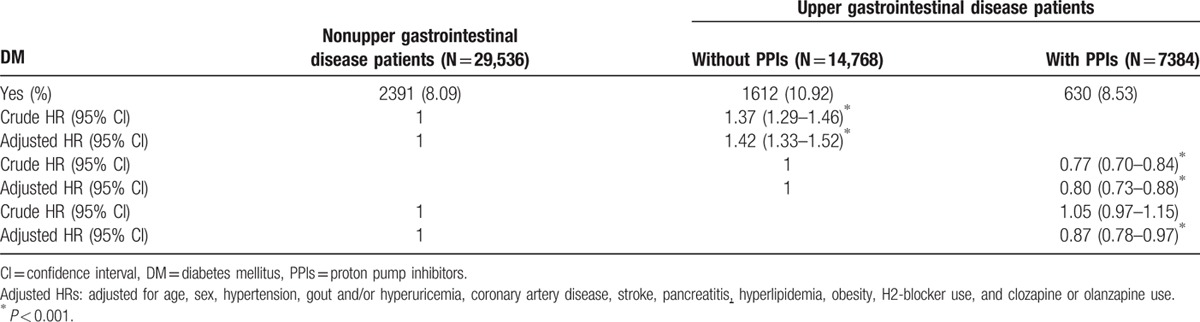
Crude hazard ratios (HRs) and adjust HRs for the risk of diabetes mellitus (DM) among the 3 patient groups.

**Figure 2:**
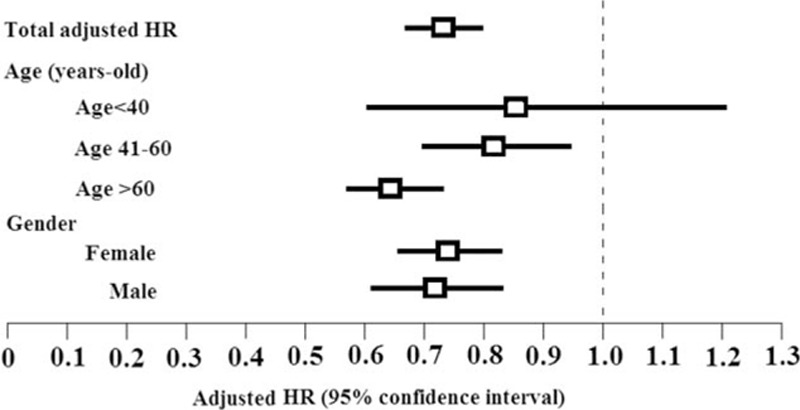
The risk of diabetes mellitus between patients with upper gastrointestinal disease (UGID) with PPI use and UGID patients without PPI use. Adjusted hazard ratios: adjusted for age, sex, hypertension, gout and/or hyperuricemia, coronary artery disease, stroke, pancreatitis, hyperlipidemia, obesity, H2-blocker use, and clozapine or olanzapine use.

Among patients receiving PPIs, those who were older than 60 years of age had a lower risk of DM (HR, 0.73; 95% CI, 0.63–0.83) than those who were younger than 40 years. Male and female patients receiving PPIs had similarly decreased risks of DM. Adjusted HRs were 0.81 (95% CI, 0.71–0.91) and 0.80 (95% CI, 0.69–0.93) for males and females, respectively (Fig. 2). We evaluated the effects of cumulative DDD on the risk of DM and observed a significant dose-related effect (*P* for trend, *P* < 0.001; Table 3). A significant increase in the onset time of DM was observed in patients who received more than 180 DDDs (816 ± 493 days) of PPIs. Patients who received >540 DDD of PPIs had the greatest reduction in the risk of DM (adjusted HR, 0.22; 95% CI, 0.14–0.35).

**Table 3:**
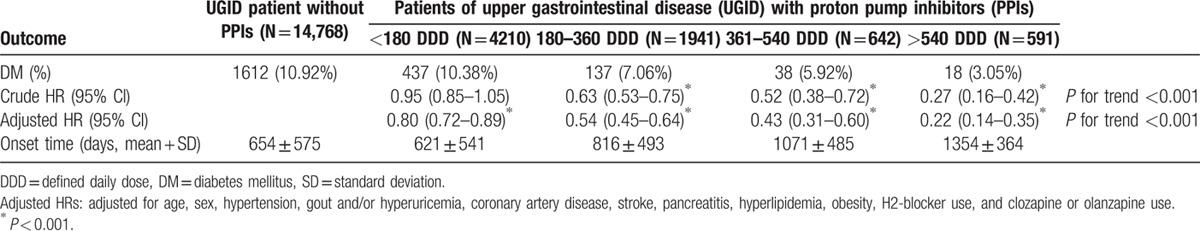
Dose effect analysis of the risk of diabetes mellitus (DM) in patients with upper gastrointestinal disease (UGID) who received proton pump inhibitors (PPIs).

**Figure 3:**
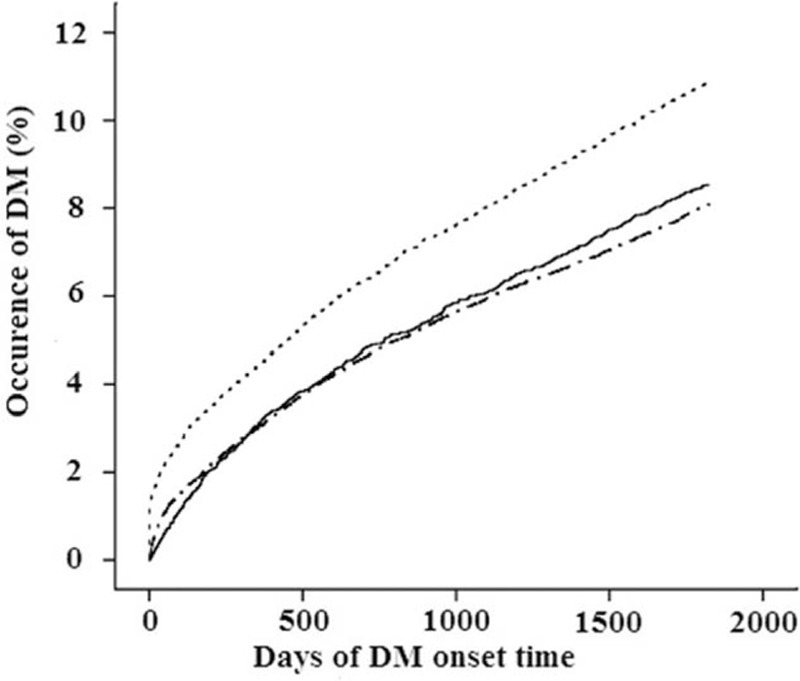
Development of diabetes mellitus among patients with upper gastrointestinal disease (UGID), patients without UGID (non-UGID), patients with proton pump inhibitor (PPI) use, and patients without PPI use. Non-UGID patients, UGID patients without PPIs, UGID patients with PPIs. Log-rank test: *P* < 0.001.

The Kaplan–Meier curve of the rate of development of DM revealed that UGID patients who did not receive PPIs had the highest cumulative incidence of DM compared to the other 2 groups (Fig. 3).

## 4 Discussion

This study is the first retrospective cohort study evaluating the effect of PPIs on DM risk in patients with UGID. We found that patients with UGID had an increased risk of DM compared to non-UGID patients, but we also found that UGID patients who received PPI therapy had a significantly lower risk of developing DM within 5 years than patients who did not receive PPIs. Furthermore, we observed a dose-related effect of PPIs on DM risk.

One possible explanation for the findings of this study is the mechanism of PPIs, which elevate intragastric pH and increase gastrin concentration.^[14]^ Suarez-Pinzon et al^[15]^ demonstrated that gastrin induced the formation of new β-cells and increased insulin secretion. Several animal studies also showed that treatment with gastrin induced the formation of new β-cells under various conditions.^[8–10,15]^ Suarez-Pinzon et al^[15]^ further showed that the combination of epidermal growth factor and gastrin increased the number of β-cells in adult human pancreatic tissue cultured in vitro and significantly increased β-cell and insulin content in human islet cells implanted in nonobese diabetic/severe combined immunodeficiency mice. Singh et al^[13]^ evaluated the effect of pantoprazole therapy on glucose-insulin homeostasis in patients with T2DM and showed that 12 weeks of pantoprazole therapy significantly increased gastrin and insulin levels and reduced HbA1c levels. Additionally, many clinical studies have commented on the beneficial effects of PPIs on glycemic control in patients with DM. Results of several studies have shown a significant reduction in HbA1c in patients with DM who were taking PPIs.^[11–13]^ Therefore, we hypothesized that UGID patients receiving PPIs may experience a decreased risk of DM. The proposed mechanism for this reduction is that PPIs increase gastrin secretion and gastrin induces islet β-cell neogenesis. Furthermore, gastrin and the related incretin hormones are both gastrointestinal peptides, so PPIs could lower glycemia through a mechanism similar to incretin-based therapies. Like incretins, gastrin increases the amount of insulin released from the β-cells of the islets of Langerhans after eating.^[16,17]^ Additionally, PPIs slow gastric emptying time, which could decrease postprandrial hyperglycemic excursions.^[18]^

This study has several noteworthy strengths. First, the Taiwan National Health Insurance is a large, population-based database which includes data from a longitudinal cohort. The nationwide Longitudinal Health Insurance Database provided an excellent resource offered a good opportunity to explore the relation between the use of PPIs and risk of DM. Second, we only included newly diagnosed UGID patients without prior DM and these patients with at least 3 consecutive episodes of diagnosed UGID. We could avoid the influence of unknown treatment histories before the study initiated and increase the accuracy of the diagnosis, respectively. Third, we took potential confounding factors for DM into consideration in the regression models. These included including age, gender, hypertension, gout or/and hyperuricemia, coronary artery disease, stroke, pancreatitis and hyperlipidemia, obesity, H2 blockers use, and clozapine/olanzapine use. Last, the further classification of PPIs users according to the dose (defined daily dose) used by the patients, demonstrating an association between larger doses of PPIs and a greater reduction of DM risk.

Nevertheless, there are some several limitations in this study, include the use of an administrative database that lacked records of patient lifestyles (such as smoking and alcohol use) and nonprescription medications use. We could not evaluate the impact of these factors. Besides, the Longitudinal Health Insurance Database is belonged to secondary database hence we could not get patients’ the body weight or body mass index. This factor is our study limitation; however, we added “obesity” as a confounding factor.

Our findings indicated that PPIs may decrease the risk of DM and with a dose-dependent effect. Further studies are warranted to determine whether PPIs have the potential to be used clinically as new antidiabetic drugs and prevention agents of DM.

We conclude that the risk of DM is increased in patients with UGID, but the risk of DM can be decreased in patients with UGID receiving PPIs. Further, the decreased DM risk in UGID patients with PPIs use is significantly dose dependent.
